# Lateral Meningomyelocele in a Neonate: A Case Report

**Published:** 2016-01-01

**Authors:** G Raghavendra Prasad, TM Rashmi

**Affiliations:** Department of Pediatric Surgery Deccan College of Medical Sciences & Princes Esra Hospital, Hyderabad, India

**Keywords:** Lateral meningomyelocele, Tethered cord, Neuro-enteric cyst, Spina bifida

## Abstract

Lateral presentation of meningocele and meningomyelocele is extremely rare. Most of the lateral meningocele described are associated with other syndromes. Isolated lateral meningomyelocele cases are rarer still. We herein report a neonate with isolated lateral gluteal meningomyelocele.

## CASE REPORT

A full-term baby boy was referred for a congenital gluteal swelling on the second day of life. The child was otherwise healthy without any neurological deficits. Examination revealed soft, cystic, fluctuant, right upper medial gluteal region (Fig. 1a,b). The swelling was reducible on pressure and refilled on release. Transillumination was suggestive of clear fluid. A plain radiograph showed classical spindle-shaped widening of interpeduncular space in the sacral region. CECT performed showed widened interpeduncular distance at S1-S3 areas, and a lateral swelling showing evidence of CSF (Fig. 1 c,d,e). Exploration revealed a CSF filled meningomyelocele with some neural elements (Fig. 1f). Neural placode was reduced and repaired with a gluteal muscle cover (Fig. 1g,h). Post-operative period was uneventful. One and a half years later, MRI revealed tethered cord with a cystic component at sacral region. The cyst was excised in toto and de-tethering of the cord was done. Post-operative period was uneventful. Histopathology of excised mass revealed glandular columnar epithelium along with some neural element. On 8 year follow-up child is doing well without any neurological sequel.

**Figure:**
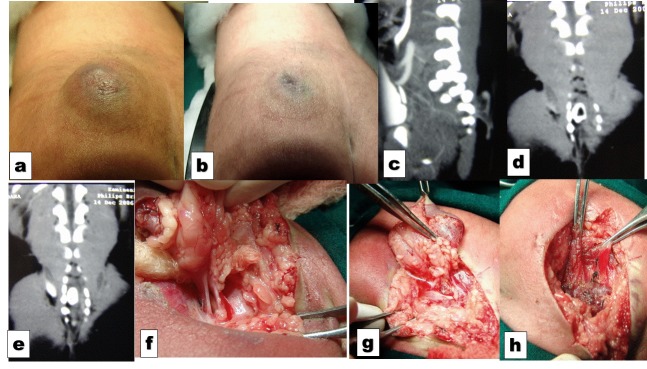
Figure 1: (a) Upper gluteal non tender soft cystic fluctuant reducible mass. (b) Collapsed on pressure. (c) Sagital view of CT scan showing the meningomyelocele. (d) Spindle shaped widening of interpeduncular space of sacrum with a defect in the bone laterally. (e) Coronal CT showing widened spinal canal with body fur. (f) Neural material seen with the margin of the defect. (g) Exteriorized neural placode in progress. (h) Gluteal flap to cover the dual defect that is closed underneath.

## DISCUSSION

Meningomyelocele are the most common forms of neural tube defects. Lateral presentations are extremely rare. Perusal of literature revealed only 2 such cases published earlier [1, 2]. Both these cases have been in older children. The index case is a neonate presenting with a compressible gluteal mass. Lateral meningocele have also been reported with other neurocutaneous syndromes and Marfans syndrome. The index case did not have any café au lait spots or multiple neurofibromas. Panil Kumar et al [3] published multiple level lateral meningocele but associated with other syndromes. Neonatal isolated gluteal meningocele has not been published earlier. Shore [4] described lateral cervical meningocele. Many meningomyelocele often are associated with tethered cord. Index case also presented at 2 years of age with tethered cord due to neuro-enteric cyst.

## Footnotes

**Source of Support:** Nil

**Conflict of Interest:** Nil
